# Supplementary material for: Serum-Derived Extracellular Vesicles from African Swine Fever Virus-Infected Pigs Selectively Recruit Viral and Porcine Proteins
Source: Viruses. 2019 Sep 20;11(10):882. doi: 10.3390/v11100882 (PMC6832119; doi:10.3390/v11100882)
Supplement: Supplementary file 1 [file viruses-11-00882-s001.pdf]

viruses

MDPI

Table 1. swine proteins identified as differentially expressed at 24dpi in OURT 88/3 infected animals.

Gene name	Protein ID	Protein Name	-Log p-value control vs A_24DPI	Difference control Vs A_24DPI
F8	K7GL28	Coagulation factor VIII	2.123919902	5.42533493
PPBP	F1RUL6	C-X-C motif chemokine	3.219079808	4.493174871
SDPR	I3LDR9	Caveolae associated protein 2	2.191007299	4.085711161
IGHG	L8B0X5	IgG heavy chain	2.084611488	-4.282530149
LOC100517145	F1S3H9	Complement C3 (LOC100517145)	3.885740476	-4.364484406
GOLM1	F1S4I1	Golgi membrane protein 1	1.746130664	-4.767168681
FCN2	I3L5W3	Ficolin-2	2.937884686	-6.029483795

Table 2. swine proteins identified as differentially expressed at 7dpi in Benin ΔMGF infected animals.

Gene name	Protein ID	Protein Name	-Log p-value control vs B_7DPI	Difference control Vs B_7DPI
	A0A075B7I5	Ig-like domain-containing protein	1.765578164	-3.480728149
ATP5A1	F1RPS8_PIG	ATP synthase subunit alpha	2.270386995	3.270935059
LOC100627396	F1RX35_PIG	Fibrinogen C-terminal domain-containing protein	2.211242648	3.967363358
LOC100514666;LOC102158263	F1RX36_PIG	Fibrinogen alpha chain	2.337934993	3.758180618
FGB	F1RX37_PIG	Fibrinogen beta chain	2.411948004	4.03753376
PSMA8	F1SBA5_PIG	Proteasome subunit alpha type	1.473601007	-3.815182686
ACAN	F1SKR0_PIG	Aggrecan core protein	1.974489764	-3.726634026
TFG	F1SL01_PIG	PB1 domain-containing protein	1.809215274	-3.131304741
LOC100154408	F1SSL6_PIG	Proteasome subunit alpha type	1.701949053	-3.944885254
PSMA4	F2Z528_PIG	Proteasome subunit alpha type	2.045768185	-4.502977371
PSMA5	F2Z5K2_PIG	Proteasome subunit alpha type	2.092257147	-4.195167542
PSMA1	F2Z5L7_PIG	Proteasome subunit alpha type	1.693446182	-4.311361313
PSMA6	F2Z5N0_PIG	Proteasome subunit alpha type	2.256749322	-4.110965729
FCN2	I3L5W3_PIG	Ficolin-2	1.114632321	-4.138368607
PSMB1	I3LQ51_PIG	Proteasome subunit beta type-1	1.236352935	-4.319245338
PSMA7	I3LVJ7_PIG	Proteasome endopeptidase complex	1.592209066	-3.87624073
GGT1	GGT1_PIG	Glutathione hydrolase 1 proenzyme	2.481172311	3.34087944
ATP2A3_tv1	K9IW69_PIG	Calcium-transporting ATPase	1.192677349	4.422147751
IGHG	L8B0X5_PIG	IgG heavy chain	1.21353349	-4.868232727
LGALS3BP	M3V7X9_PIG	Lectin, galactoside-binding, soluble, 3 binding protein	0.933748348	-5.950680733
PPBP	CXCL7_PIG	Platelet basic protein	1.81832725	3.661399841

	Q28936_PIG	Fibrinogen A-alpha-chain	2.66797977	4.091690063

Table S3. swine proteins identified as differentially expressed at 24dpi in Benin ΔMGF infected animals.

Gene name	Protein ID	Protein Name	-Log p-value control vs B_24DPI	Difference control Vs B_24DPI	
ACDC	Q6V9B4	Adiponectin	1.68103827	-3.785552979	
AOC3	F1S1G8	Amine oxidase	1.945752564	-3.751467133	Cell adhesion protein that participates in lymphocyte extravasation and recirculation by mediating the binding of lymphocytes to peripheral lymph node vascular endothelial cells in an L-selectin-independent fashion
ATP5A1	F1RPS8	ATP synthase subunit alpha	2.847389826	3.819492817	
ATP5B	Q0QEM6	ATP synthase subunit beta	1.739728871	3.515419006	
ATP2A3_tv1	K9IW69	Calcium-transporting ATPase	2.223654113	4.816053391	
PRKAR1A	P07802	cAMP-dependent protein kinase type I-alpha regulatory subunit	1.288915259	4.504089355	
CAVIN2	I3LDR9	Caveolae associated protein 2	1.354488605	5.447482745	
F8	K7GL28	Coagulation factor VII	3.02428948	5.472725296	
CORO1C	F1RGA9	Coronin	1.391760825	3.911675453	
PPBP	F1RUL6	C-X-C motif chemokine	2.657114869	5.152716955	This growth factor is a potent chemoattractant and activator of neutrophils
EMILIN1	F1SDQ5	Elastin microfibril interfacer 1	1.907341643	4.285336018	
FERMT3	F1RQ01	Fermitin family member 3	2.748417874	3.717049599	Kindlins are a small family of proteins that mediate protein-protein interactions involved in integrin activation and thereby have a role in cell adhesion, migration, differentiation, and proliferation. The protein encoded by this gene has a key role in the regulation of hemostasis and thrombosis
N/A	Q28936	Fibrinogen A-alpha-chain	2.342393148	4.619222005	
FGB	F1RX37	Fibrinogen beta chain	2.431243123	3.964152972	
FN1	F1SS24	Fibronectin 1	2.250441913	-3.020938555	

FCN2	I3L5W3	Ficolin-2	2.274392861	-5.66368707	May function in innate immunity through activation of the lectin complement pathway. Calcium-dependent and GlcNAc-binding lectin
GGT1	P20735	Glutathione hydrolase 1 proenzyme	1.736149213	3.669027328	
GPIbA	B6ECP2	Glycoprotein Ib platelet alpha subunit	2.681522725	3.341304588	
GPIIb	Q9TUN6	Glycoprotein IIb	3.917758116	4.045817693	
GOLM1	F1S4I1	Golgi membrane protein 1	2.28597367	-4.857357025	
HSPA1B	Q6S4N2	Heat shock 70 kDa protein 1B	1.974503015	3.234610558	
IGHG	L8B180	IgG heavy chain	2.373289221	-3.076951027	
IGHG	L8B0W5	IgG heavy chain	2.000232471	-3.521834691	
IGHG	L8B0X5	IgG heavy chain	3.60062972	-5.399693807	
CD61	D6BR76	Integrin beta	2.180958059	3.606406848	
ITGB1	F1RVE7	Integrin beta	3.972272372	2.923595746	
ILK	I3L9C8	Integrin linked kinase	1.988120564	4.824102084	
ITGA6	K7GT68	Integrin subunit alpha 6	1.749338143	4.950547854	
ITIH1	F1SH96	Inter-alpha-trypsin inhibitor heavy chain H1	2.356516145	-3.534379196	
JCHAIN	F1RUQ0	Joining chain of multimeric IgA and IgM	2.174132721	-3.966109276	
LY6G6	A0A1L6ZA05	Lymphocyte antigen 6 complex locus G6F	1.826127125	3.890546799	G6f protein is a type I transmembrane protein belonging to the immunoglobin (Ig) superfamily, which is comprised of cell-surface proteins involved in the immune system and cellular recognition
MYH9	K9IVP5	N-myosin-9	2.648360079	3.367460569	
PGAM1	F1S8Y5	Phosphoglycerate mutase	3.006333036	4.45399793	
PLEK	F1SJ07	Pleckstrin	1.857273097	4.696280479	
PSMA1	F2Z5L7	Proteasome subunit alpha type	2.338316455	-3.325544357	
PSMA5	F2Z5K2	Proteasome subunit alpha type	2.851858881	-3.328734716	
RAP1A	I3L5L1	RAP1A, member of RAS oncogene family	2.781650335	3.057108879	
STXBP2	F1SCI9	Syntaxin-binding protein 2	1.886460976	3.653300285	Involved in intracellular vesicle trafficking and vesicle fusion with membranes. Contributes to the granule exocytosis machinery through interaction with soluble N-ethylmaleimide-sensitive factor attachment protein receptor (SNARE) proteins that regulate membrane

					fusion. Regulates cytotoxic granule exocytosis in natural killer (NK) cells
THBS4	F1RF28	Thrombospondin 4	1.652059272	-3.914692879	
TUBA4A	F2Z5S8	Tubulin alpha chain	2.185756575	3.549171448	
TUBB	Q767L7	Tubulin beta chain	1.933344877	3.510186513	
TUBB1	A5GFX6	Tubulin beta chain	2.279609551	3.379505157	
ITGA2	K7GPK0	Uncharacterized protein	2.124166667	4.362858772	Loss of the encoded protein is associated with bleeding disorder platelet-type 9
FGG	F1RX35	Uncharacterized protein	2.747714093	4.054901441	This protein is important for blood clot formation (coagulation), which is needed to stop excessive bleeding after injury
LOC100514666	F1RX36	Uncharacterized protein	2.717573188	4.01283741	
MYL12B	F2Z5V6	Uncharacterized protein	2.763041986	3.532177544	
DELETED	I3LS04	Uncharacterized protein	1.660923531	3.393268585	
C4BPA	F1S0J2	Uncharacterized protein	1.775641255	-3.30101649	
PSMB1	I3LQ51	Uncharacterized protein	1.601525029	-3.579036713	The 20S proteasome mediates ubiquitin-independent protein degradation. This type of proteolysis is required in several pathways including generation of a subset of MHC class I-presented antigenic peptides
SERPING1	F1SJW8	Uncharacterized protein	1.551996553	-3.582340876	C1 inhibitor is important for controlling a range of processes involved in maintaining blood vessels, including inflammation. Inflammation is a normal body response to infection, irritation, or other injury
LOC100517145	F1S3H9	Uncharacterized protein	2.822347213	-4.074431229	
DELETED	A0A075B7I7	Uncharacterized protein	2.567990759	-4.125251452	
ITIH3	F1SH94	Uncharacterized protein	2.35014255	-4.13372612	
N/A	F1STC2	Uncharacterized protein	2.585282992	-4.145962079	
CD5L	F1RN76	Uncharacterized protein	2.057836202	-4.201869329	
WDR1	K9IVR7	WD repeat domain 1	2.478896272	3.582942327	
